# Vitamin D Deficiency and Insufficiency According to Current Criteria for Children: Vitamin D Status of Elementary School Children in Turkey

**DOI:** 10.4274/jcrpe.galenos.2019.2019.0103

**Published:** 2019-09-03

**Authors:** Ahmet Anık, Özgür Akbaba

**Affiliations:** 1 Aydın Adnan Menderes University Faculty of Medicine, Department of Pediatric Endocrinology, Aydın, Turkey; 2 Beşiktaş No 3 Family Health Center, İstanbul, Turkey

## Dear Editor,

We read the article of Hocaoğlu-Emre et al (1) entitled ‘Vitamin D Deficiency and Insufficiency According to the Current Criteria for Children: Vitamin D Status of Elementary School Children in Turkey’ in the Journal of Clinical Research in Pediatric Endocrinology with great interest. In this study, the researchers investigated serum vitamin D levels in 640 healthy children between the ages of 6 and 9 years. It was stated that serum vitamin D levels of the subjects were obtained from the hospital records. They explained further that vitamin D levels were checked in healthy children by an “annual check-up for vitamin D status” at the hospital. The authors conclude that close follow-up of vitamin D status especially in the winter and post-winter period is necessary and that vitamin D supplementation be given for a strong bone structure and healthy growth (1).

Vitamin D deficiency screening should aim to identify people with low vitamin D levels who theoretically could benefit from vitamin D supplementation. Only after this theoretical screening program, we would expect improvement in particular health outcomes e.g improved bone mineral density, reduced risk of falls etc. Furthermore in any screening programme, the intervention and subsequent treatment should be harmless (2). However, there is no firm evidence showing benefits of vitamin D deficiency screening for healthy children (3,4). Recent global consensus recommendations caution strongly against population-based screening for vitamin D deficiency in healthy children (3). According to this consensus, serum 25(OH)D measurement would be reasonable for patients with high risk of vitamin D deficiency, such as patients having rickets, chronic kidney disease, hepatic failure, malabsorption, hyperparathyroidism or granuloma-forming disorders (3). Similarly, the American Academy of Pediatrics advises screening only in patients who have disorders associated with low bone mass such as rickets and/or a history of recurrent, low-trauma fractures (4). In addition, there has been a significant increase in health costs related to vitamin D tests and prescriptions for children in primary care over the past decade (5).

In conclusion, current evidence is not sufficient to suggest that screening for vitamin D deficiency in a healthy population produces health benefits, is necessary, safe or cost-effective.

## References

[1] Hocaoğlu-Emre FS, Sarıbal D, Oğuz O (2019). Vitamin D Deficiency and Insufficiency According to the Current Criteria for Children: Vitamin D Status of Elementary School Children in Turkey. J Clin Res Pediatr Endocrinol.

[2] Minisola S, Colangelo L, Cipriani C, Pepe J, Cook DP, Mathieu C (2019). Screening for hypovitaminosis D: cost-effective or not?. Eur J Endocrinol.

[3] Munns CF, Shaw N, Kiely M, Specker BL, Thacher TD, Ozono K, Michigami T, Tiosano D, Mughal MZ, Mäkitie O, Ramos-Abad L, Ward L, DiMeglio LA, Atapattu N, Cassinelli H, Braegger C, Pettifor JM, Seth A, Idris HW, Bhatia V, Fu J, Goldberg G, Sävendahl L, Khadgawat R, Pludowski P, Maddock J, Hyppönen E, Oduwole A, Frew E, Aguiar M, Tulchinsky T, Butler G, Högler W (2016). Global Consensus Recommendations on Prevention and Management of Nutritional Rickets. J Clin Endocrinol Metab.

[4] Yeşiltepe Mutlu G, Hatun Ş (2018). Use of Vitamin D in Children and Adults: Frequently Asked Questions. J Clin Res Pediatr Endocrinol.

[5] Basatemur E, Hunter R, Horsfall L, Sutcliffe A, Rait G (2017). Costs of vitamin D testing and prescribing among children in primary care. Eur J Pediatr.

